# Use of the Taguchi Method to Optimize an Immunodetection System for Quantitative Analysis of a Rapid Test

**DOI:** 10.3390/diagnostics11071179

**Published:** 2021-06-29

**Authors:** Kai-Wen Lin, Yu-Chi Chang

**Affiliations:** Department of Engineering Science, National Cheng Kung University, Tainan 701, Taiwan; kevlin.esc@gmail.com

**Keywords:** rapid test, immunodetection system, Taguchi method

## Abstract

In this research, the Taguchi method was used to optimize the detection accuracy and reproducibility of an immunodetection system used for a quantitative analysis of a rapid test. Furthermore, the standard deviation (SD) and coefficient of variation (CV) between the theoretical value and the measured value of the self-made simulated rapid test became smaller, and the linearity became higher. The results thus indicated that the immunodetection system became more reliable. In the present research, a camera was used to capture an image containing the control line (C line) and the test line (T line) in the self-made simulated rapid test. The captured image was then analyzed, and the grayscales of the C line and T line were calculated. The Taguchi method was used to adjust the light intensity of the light-emitting diode (LED) and the camera parameters in the immunodetection system to determine the optimal parameters by which to optimize the performance of the immunodetection system. The goal of the present research was to obtain a measurement with a minimum SD and CV between the detected grayscales and the grayscales of the self-made simulated rapid test, thus indicating successful development of a practical, stable, and accurate immunodetection system. To mimic the color expression in an actual rapid test, the ratio of the red, green, blue (RGB) components of the self-made simulated rapid test had to be adjusted to closely fit the color expression of the actual rapid test. After the RGB ratio was set, the Taguchi method was used to optimize the parameters for the purpose of detection. When the optimal parameters were found, the signal-to-noise ratio (*S*/*N* ratio) had been increased from −12.89 dB to −10.91 dB, which means the accuracy of the color detection had been improved. Compared to the original detection system, the quality loss had been reduced to 33.1%.

## 1. Introduction

Lots of different technologies are used for immunodetection; for example, millions of transducers are fabricated on a chip to detect or screen thousands of analytes by adapting magneto-resistive computer memory technology. A prototype of the giant magnetoimpedance (GMI)-based biosensing system has been set up to broaden its application for targeted detection of gastric cancer cells by measuring magnetoimpedance (MI) responses [1,2,3].

Colloidal gold immunochromatography is a commonly used immunoblotting technique in clinical practice. Nanoparticles with diameters ranging from 1 to 150 nm are in a dispersed phase. Depending on the size of the nanoparticles, they can appear in terms of color from orange-red (2–20 nm) to purple-red (30–80 nm). Colloidal gold with specific antibodies can be conjugated to form a stable antibody-AuGP (gold nanoparticles) conjugation. Because it combines the stability and specificity of antibodies, coupled with the clear coloring of colloidal gold particles, it is possible to produce stable, rapid, specific, and sensitive test reagents [4,5,6].

A lateral flow immunochromatographic analysis is one of the important methods used in modern rapid biological detection [7,8]. The principle is to use the specificity and immunoaffinity between the antigen and an antibody as the basis for detection, where colloidal gold is used as the chromogenic agent.

When the concentration of the targeted specimen increases, more target specimens are captured. This causes more colloid gold aggregation and increases the color density, which is used as a basis for interpretation.

The major disadvantages of the enzyme-linked immunosorbent assay (ELISA) include the fact that it is labor-intensive, has a high possibility of false positive/negative results, and refrigerated transport and storage are required for the antibodies [9].

Colloidal gold immunochromatography is commonly used for rapid detection. Even though an immunoassay based on lateral flow operates on the same principle as the ELISA, it has many advantages, including the fact that it is inexpensive, delivers fast results (within 5 to 15 min), and does not require processing in a laboratory. Therefore, it is superior to the conventional ELISA and has relatively good sensitivity reproducibility [10,11,12,13]. It is widely used in clinical testing and agricultural product testing, in drug testing in the military and police agencies, and in environmental protection.

The color immunoassay is very practical in the field of immunodetection. More scientific methods can be used, such as taking the image and submitting it to the program in order to use the image result as the criterion for a quantitative determination intended to improve the detection sensitivity and the objectivity of the test results [14]. Therefore, to meet clinical requirements, there is a need for quantitative detection of targeted specimens, so the development of rapid tests and image detection equipment thus has broad appeal and potential. Image-based immuno-chromatographic test strip readers have already been demonstrated by a few researchers. In 2011, Mei et al., designed and fabricated a new lateral flow test strip reader operating system based on an Advanced RISC Machine (ARM) processor and an embedded Linux based software. The instrument uses a green light-emitting diode as the light source, where it captures the test strip image with a high-resolution complementary metal oxide semiconductor (CMOS) image sensor, and then the image-processing method is used to calculate the result [15]. In 2012, Mudanyali et al., demonstrated a cellphone-based rapid-diagnostic-test (RDT) reader platform that works with various lateral flow immuno-chromatographic assays and similar tests to sense the presence of a target analyte in a sample [16]. In 2015 and 2016, Chowdhury et al., developed a reader for quantitative lateral flow immunoassays using a line laser diode module to illuminate a lateral flow immunoassay test strip with fluorescent dye [17,18].

The purpose of the present research is to use Taguchi method to optimize the immunodetection system used for the lateral flow chromatography rapid test so that the immunodetection system has a standard calibration curve with high linearity and a low standard deviation (SD) at a specific concentration, and the quantitative analysis results are more accurate. The Taguchi method is a statistical tool, which through the process parameter levels and the experimental plan, simplifies the number of experiments and improve the method used for data analyses. It can be applied to engineering [19,20,21], manufacturing [22,23], and the supply chain [24].

In 2019, Majeed et al., validated measurement and calculation condition parameters using the Taguchi method to prove that the secure force cryptographic method is lightweight and provides better image quality. The jpg images were validated to have better image quality parameters for security purposes [25].

In 2020, Zhang et al., evaluated the influence of each thermal design parameter on imaging quality based on an integrated thermo-optical analysis. Then, they applied the Taguchi method to quantitatively calculate the effect of each thermal design parameter on image quality. Finally, with the results of the Taguchi method, the optimal thermal control design of a space camera was determined [26].

In research, there are four significant factors that affect the results of image detection in an immunodetection system, including the light intensity of the light source in the optical darkroom, the contrast, the saturation, and the tone of the camera. We used the Taguchi method to find the optimal parameters so that the immunodetection system has a better signal-to-noise ratio (*S*/*N* ratio), a smaller deviation, and higher linearity.

## 2. Experimental Procedure

### 2.1. Experimental Principles

**Figure 1:**
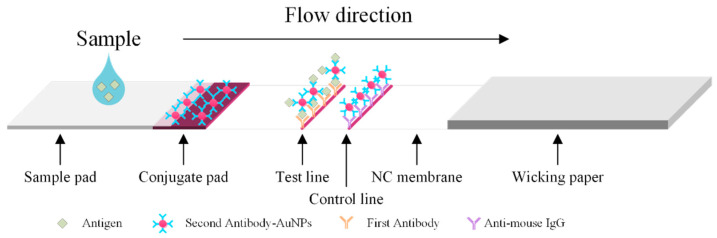
Principle of the lateral flow test.

A rapid test involves the use of a lateral flow chromatographic immunoassay to detect a target antibody or antigen. The rapid test is made of four major materials, including a sample pad, a conjugate pad, nitrocellulose (NC) membranes, and wicking paper. Lateral chromatography uses NC membrane with large-pore micropores. The NC membrane is the carrier, with the specific antigen or antibody fixed on it. When the sample to be tested is added to the sample pad in the test strip, it moves laterally due to capillary action and reacts specifically with the labeled antibody on the conjugate pad. It then migrates on the NC membrane toward the wicking paper. When the targeted antibody or antigen is captured, the aggregated gold-nanoparticles (AuGP) express a red-purple color, while the unbound antibody/antigen-AuGP conjugates flow through the detection zone without exhibiting color. An illustration of a rapid test is provided in Figure 1.

When the targeted antibody/antigen concentration increases, the quantity of the conjugates of the targeted antibody/antigen and AuGP increases as well. This results in the color of the test line being stronger. The color intensity can be captured and analyzed using an immunodetection system, and then can be output as a quantitative result. To establish a strong relationship between the theoretical value and the measured value of the self-made simulated rapid test proposed in this work, the SD and coefficient of variation (CV) have to be decreased, and the linearity has to be increased through experimental adjustment of the light intensity of the light-emitting diode (LED) and the camera parameters via the use of the Taguchi method.

### 2.2. Hardware Platform

**Figure 2:**
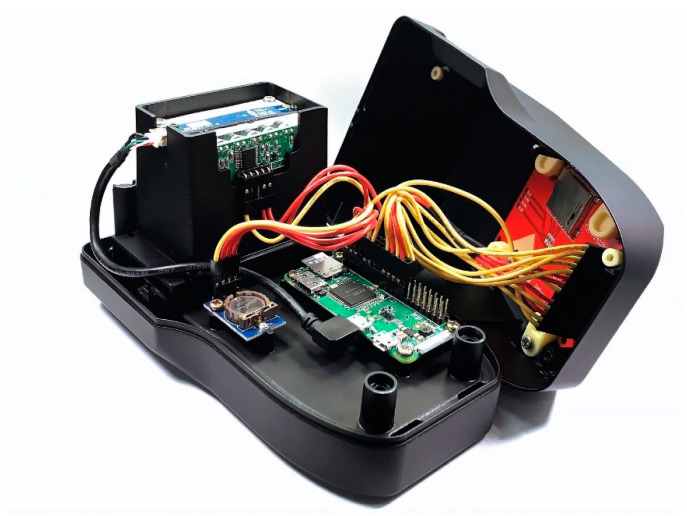
The immunodetection system.

**Figure 3:**
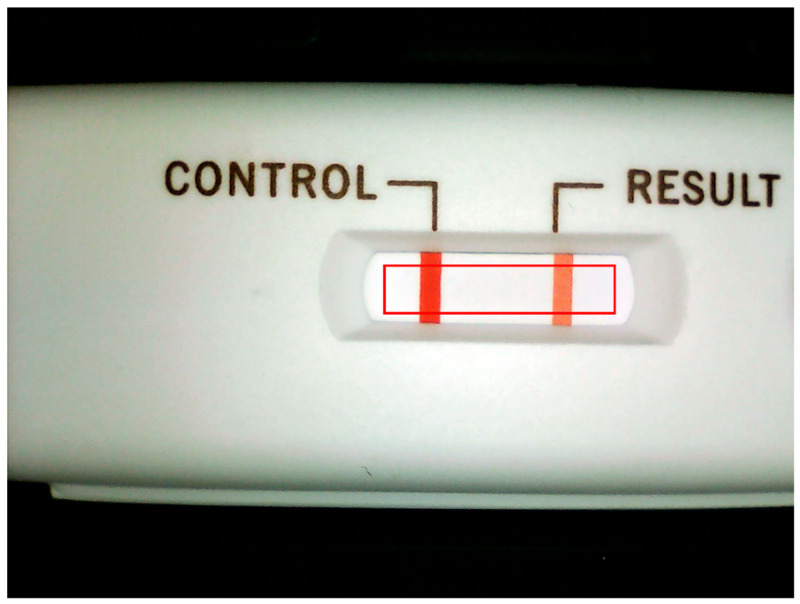
Captured image of the cassette with the region of interest (ROI) area marked with a red line.

The exterior design and internal mechanism layout of the immunodetection system were designed by SolidWorks. The immunodetection system is shown in Figure 2. The system includes a housing, an optical darkroom, and a rapid test cassette carrier. The function of the optical darkroom is to prevent the test environment from being affected by external light. The optical darkroom comprises a Universal Serial Bus (USB) camera, a light guide plate, and an LED circuit board. The USB camera captures an image of the specific area containing the control line and test line on the rapid test. The image is then analyzed to obtain the grayscale values of the control line (C line) and test line (T line), as shown in Figure 3.

### 2.3. Experimental Procedure

The colors of the C and T lines for the actual rapid tests were derived from aggregated colloidal gold. The coloration changes with the concentration of the colloidal gold nanoparticles in the captured target. To avoid uncontrollable color variations caused by the actual rapid tests when using the Taguchi method to optimize the parameter—since this will affect the results of the experiment—the self-made simulated color strips were made to match the actual colors of the C and T lines, which range between purple and red, as the measurement targets.

**Figure 4:**
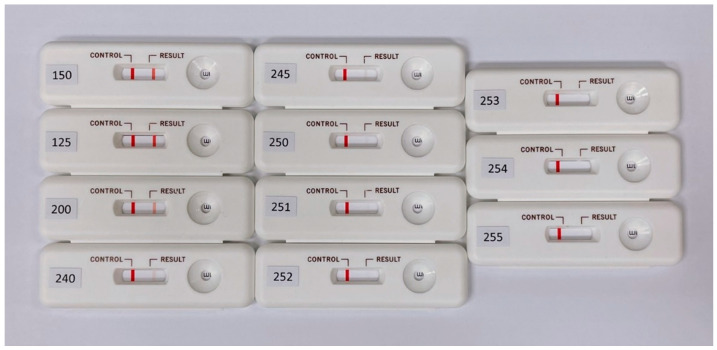
Designed self-made simulated rapid test strips with different grayscale values ranging from 125 to 255 for the T line.

When quantifying the color rendering using commercial software, the primary color of the RGB distribution of an actual immune test strip is close to red. The R-values in the three primary RGB colors in the self-made simulated rapid test were fixed, and the values of G and B were changed to design the tests with different grayscale values ranging from 125 to 255. The designed self-made simulated rapid test strips are shown in Figure 4.

The Taguchi method was used to design the experiments used to investigate four different parameters affecting the detection results. Four parameters with obvious influences on the results were selected based on experience, including the light intensity (control factor A), contrast (control factor B), color saturation (control factor C), and tone (control factor D). The L_9_(3^4^) Taguchi orthogonal arrays were chosen to organize the parameters affecting the detection results, since their levels should vary. The four parameters at three levels are shown in Table 1.

Because the relationship between the measured values and the input color levels is linear, the dynamic quality characteristic is chosen as the quality characteristic, and Equation (1) for the *S*/*N* ratio is shown as follows:

(1) $$S/N_{SB}=-10\log\left(\frac{S_{d}^{2}}{\beta^{2}}\right),$$

where *S_d_* is the dynamic standard error used to estimate the deviation between the experimental data and the regression equation, and *β* is the slope. The correlation of the theoretical value and the measured values for the self-made simulated rapid test strip is expected to exhibit linear dependence with a correlation coefficient close to 1. An immunodetection system was used determine the grayscale value of the self-made simulated rapid test strip and establish a linear relationship between the theoretical value and the measured grayscale value of the self-made simulated rapid test strip.

## 3. Results and Discussion

### The Taguchi Approach

Based on the L_9_(3^4^) Taguchi orthogonal arrays, changes in the levels of the control factors can be used calculate the influence of each control factor, predict their optimum levels, and verify their performance.

**Figure 5:**
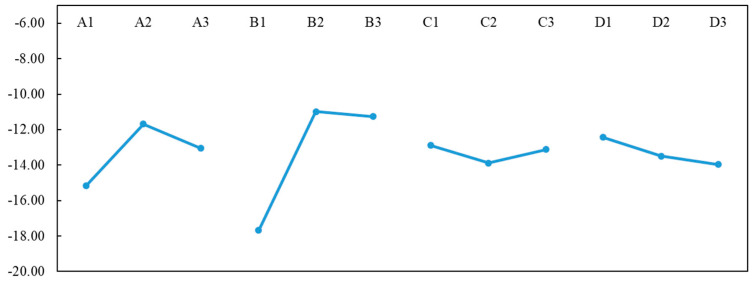
*S*/*N* response table for the four control factors of interest, where A is light intensity, B contrast, C color saturation, and D tone.

When the *S*/*N* ratio values are calculated with each factor and at each level, the R (R = high *S*/*N*–low *S*/*N*) value of the *S*/*N* for each factor is calculated and entered into the response chart. A larger R value for a factor indicates that the factor level has a greater influence on the process. The factor response table for the *S*/*N* ratio is shown in Table 2 and Figure 5. In Table 2, it can be seen that the control factors and levels affect the interpretation of the grayscale values, where the influence of B is more significant than that of the other factors. The original design control factors were A3, B2, C2, and D2. According to the larger-the-better *S*/*N* function, the optimal parameters of the control factors become A2, B2, C1, and D1. If the analysis is conducted based on the one-half criterion, the factors A and B have the greatest influence.

However, this method of comparing the magnitude of significance is a very basic judgment. Some factors may be insignificant. They can even be regarded as an experimental deviation, or they may not be statistically meaningful. If the insignificant factors are included in the calculation of the prediction, the total factor effect will be overestimated and in turn will make the confirmation experiment unable to match the predicted value. Therefore, before confirming the optimal parameters of the control factors, it is necessary to evaluate which control factors cause experimental deviations. In other words, the insignificant factors need to be determined and excluded from the calculation of the prediction. A further statistical analysis is needed to evaluate the deviations, and then the experiments can be analyzed using an analysis of variance (ANOVA) to determine the important factors. The important factors then are used to design the optimal experiments to obtain the correct predicted values of the important factors, which will then be used to perform the verification experiments.

In an ANOVA, when the confidence level of the control factors is higher than 95%, the control factors have a sufficient influence on the *S*/*N* ratio. It can be seen from Table 3 that the confidence levels for control factors A and B are greater than 95%, which means that these two control factors have a greater influence on the *S*/*N* ratio control factors C and D. Therefore, in the confirmation experiments, only two factors, A (Light intensity) and B (Contrast), were selected to be changed; the factors C (Color saturation) and D (Tone) remained unchanged, where the optimal parameters for the control factors were A2, B2, C2, and D2.

**Figure 6:**
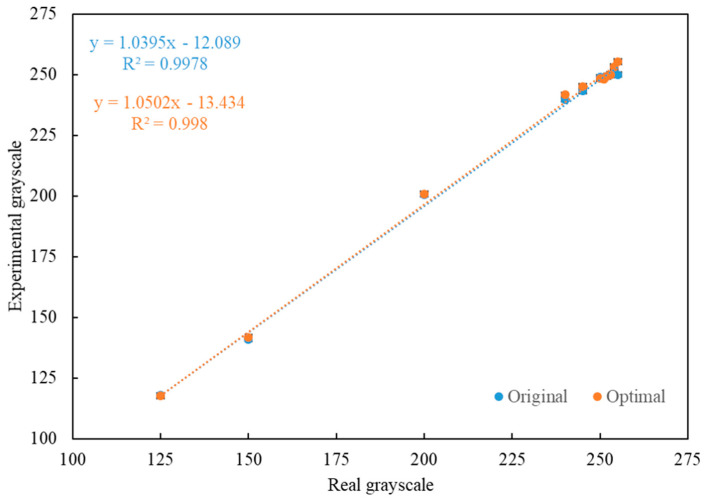
The relationship between the original design and the optimal design of the self-made simulated rapid test strip.

The final step of the Taguchi method is to use the optimal control factor parameters selected from the results of the ANOVA to calculate the predicted *S*/*N* ratios for the original design and the optimal design, and compare the *S*/*N* ratios for the experimental values obtained for the original design with those of the optimal design from the confirmation experiments in triplicate. The results are shown in Table 4 and Figure 6. In Table 4, it can be seen that the *S*/*N* ratio from the original design to the experimental value of the optimal design was increased by 1.98 dB, from −12.89 dB to −10.91 dB. Figure 6 shows that the R-squared value of the optimal design is better than the R-squared value of the original design, which means the linearity is better. According to the *S*/*N* ratios shown in Table 4 and the R-squared values shown in Figure 6, the results for the optimal design are better than the results for the original design.

The cost of a product during the entire life cycle is the quality loss, where less quality loss represents higher quality. Generally, when the calculated *S*/*N* ratio increases by 3 dB, the quality loss will be reduced to half that of the original value. It can be seen that the quality loss was reduced to 33.1% from Equation (2).

(2) $$\frac{Q_{optimal}}{Q_{original}}\approx 0.5^{\frac{S/N_{optimal}-S/N_{original}}{3}}=0.5^{\frac{-10.91+12.89}{3}}=0.331,$$

where *Q_optimal_* and *Q_original_* represents the quality loss of the design, and *S*/*N_optimal_* and *S*/*N_original_* are the *S*/*N* ratios of the design.

Since the predicted *S*/*N* ratio from the confirmation experiment was calculated from the empirical model in the Taguchi method, if the predicted *S*/*N* ratio is consistent with that of the confirmation experiment, it can be concluded that the experiment is reliable. The allowable deviation in the predicted value of *S*/*N* for the 99% confidence interval (CI) of the confirmation experiment was set at +/−5.01 dB. The difference between the predicted *S*/*N* ratio and the *S*/*N* ratio obtained from the confirmation experiment was 1.55 *dB*, which is less than the 1% allowed deviation, as shown in Equation (3), which means the experiment was reliable.

(3) $$CI=t_{\alpha }\left(f_{e}\right)\sqrt{MS_{e}\times \left(\frac{1}{m_{e}}+\frac{1}{m_{r}}\right)}=\pm 1.55dB,$$

where *α* is the significance level; $f_{e}$ is the degrees of freedom of the pooled error; $MS_{e}$ is the mean square of the pooled error; $m_{e}$ is the equivalent sample size for the confirmation experiment, and $m_{r}$ is the sample size for the confirmation experiment.

## 4. Conclusions

Through the optimal design using the Taguchi method, the *S*/*N* ratio was increased from −12.89 dB to −10.91 dB, where the overall improvement was 1.98 dB. In this experiment, it was verified that the contrast and light intensity have a significant influence on the detection results.

The 1% allowable deviation was 5.01 dB, and the deviation between the predicted *S*/*N* ratio and that of the confirmation experiment was 1.55 dB, which is much smaller than the allowable deviation of 5.01 dB. Therefore, the confirmation experiment was deemed reliable.

Comparing the R-square of the linear regression of the grayscale values of the optimized design with those of the original design, the optimized value was higher than that of the original design, which means that the linear regression of the detection results for the optimized design was more linear and better that obtained for the original design.

The immunodetection system was improved by using the Taguchi method to design the optimal experiments for adjustment of the control factors. In future, this immunodetection system can be applied to lateral flow strip inspection and color testing of different items.

## Figures and Tables

**Table 1:** Control Factors & Levels of Control Factors.

Control Factors		Level		
		1	2	3
A	Illuminance (lux)	200	240	280
B	Contrast	0.13	0.33	0.53
C	Color Saturation	0.35	0.5	0.65
D	Tone	0.35	0.5	0.65

**Table 2:** Factor Response Analysis.

	A (Light Intensity, Lux)	B (Contract)	C (Color Saturation)	D (Tone)
Level 1	−15.2	−17.7	−12.9	−12.4
Level 2	−11.7	−11.0	−13.9	−13.5
Level 3	−13.1	−11.3	−13.1	−14.0
Range	3.5	6.7	1.0	1.5
Rank	2	1	4	3

**Table 3:** Analysis of Variance for the *S*/*N* Ratios.

Factors	SS	DOF	Var	F	Probability	Confidence	Significance *
A	18.54	2	9.27	6.96	49.8%	95.02%	Yes
B	85.88	2	42.95	32.23	0.34%	99.66%	Yes
C	Pooled						
D	Pooled						
Error	5.32	4	1.33	S = 1.1542			
Total	109.76	8					

* At least 95% confidence.

**Table 4:** The relationship between the original design and the optimal design of the self-made simulated rapid test strip.

	125	150	200	240	245	250	251	252	253	254	255	*β*	*S_d_*	*S*/*N*	
														Exp.	Predicted
Original	117.85	140.91	200.59	239.81	243.31	248.93	248.61	249.19	249.81	250.64	250.04	0.9998	4.41	−12.89	−13.55
Optimal	117.68	141.76	200.83	241.71	245.05	248.37	248.21	249.35	250.05	253.29	255.28	0.9925	3.48	−10.91	−9.35

## Data Availability

Not applicable.
